# Mindful Eating and Its Relationship with Obesity, Eating Habits, and Emotional Distress in Mexican College Students

**DOI:** 10.3390/bs15050669

**Published:** 2025-05-14

**Authors:** Irina Lazarevich, María Esther Irigoyen-Camacho, Claudia Cecilia Radilla-Vázquez, Rey Gutiérrez-Tolentino, Maria Consuelo Velazquez-Alva, Marco Antonio Zepeda-Zepeda

**Affiliations:** Division of Biological and Health Science, Metropolitan Autonomous University-Xochimilco, Calzada del Hueso 1100, Col Villa Quietud, Mexico City 04960, Mexico; iboris@correo.xoc.uam.mx (I.L.); cradilla@correo.xoc.uam.mx (C.C.R.-V.); reygut@correo.xoc.uam.mx (R.G.-T.); mcvelaz@correo.xoc.uam.mx (M.C.V.-A.); mzepeda@correo.xoc.uam.mx (M.A.Z.-Z.)

**Keywords:** mindful eating, body mass index, body fat percentage, eating habits, depression, anxiety, stress, college students

## Abstract

Mindful eating (ME) has gained recognition in multidisciplinary weight management intervention and prevention programs for dysfunctional eating behaviors. This study aimed to evaluate the associations of mindful eating with body mass index, fat percentage, unhealthy food consumption, and emotional distress in Mexican college students. A cross-sectional study was performed. Anthropometry and body composition were evaluated. A self-reported Mindful Eating Questionnaire developed for the Mexican population, Food Frequency Questionnaire (frequency and serving size), and Depression, Anxiety and Stress Scale (DASS-21) were applied. Two separate analyses were conducted: the first used the whole Mexican Mindful Eating Questionnaire with 11 questions (ME-11), and the second excluded the emotional eating items (ME-8). A total of 224 students were included in the analysis. Lower levels of mindful eating were associated with higher body mass index (BMI) (*p* < 0.001), waist circumference (*p* < 0.001), and body fat percentage (*p* < 0.001), using ME-11 or ME-8. Significant associations were identified between lower levels of mindful eating (ME-11) and consumption of fried foods (*p* = 0.005), sweets and desserts (*p* = 0.003), and fast food (*p* = 0.003). Similar associations were observed using the ME-8 score. In both versions of the questionnaire, depression, anxiety, and stress scores were significantly associated with lower levels of mindful eating. Mindful eating was associated with BMI, body fat, eating habits, and emotional distress. Mindful eating could be used in multidisciplinary educational and intervention programs to promote a healthy lifestyle.

## 1. Introduction

Mindful eating (ME) has gained recognition in multidisciplinary weight management, energy intake and food choice, dysfunctional eating behaviors, and eating disorders (Beccia et al., 2018; Mantzios et al., 2018a; Sala et al., 2020, 2022; Tapper, 2022). Emotional distress, such as anxiety, stress, or depression, is frequently related to unhealthy eating behaviors. Mindful eating may help change this pattern by promoting awareness of emotional triggers and supporting a more balanced, non-judgmental response to eating (Giannopoulou et al., 2020; Metin et al., 2024; Winkens et al., 2018, 2020).

Obesity is a complex, heterogeneous, chronic, and progressive disease. Its prevalence in adults over 18 years of age significantly increased worldwide between the years 1990 and 2024 (Lingvay et al., 2024; WHO, 2024). In Mexico, the prevalence of overweight and obesity is one of the highest in the world, which is associated with the most common chronic diseases. By 2022, 22.9% of adolescents were overweight and 18.2% obese, while in the population between 20 and 39 years old, 35% were overweight and 33% obese. Additionally, a large proportion of Mexican adults had a high waist circumference, which is also an indicator of metabolic risk (Campos-Nonato et al., 2023; Shamah-Levy et al., 2022). Different studies reported that the combined prevalence of overweight and obesity in Mexican college students ranges from 27% to 49% (Cruz-Rodríguez et al., 2019; Fernández-Carrasco & López-Ortiz, 2019; Lazarevich et al., 2018; Lorenzini et al., 2015).

Factors that contribute to weight gain include overconsumption of food and low physical activity, which, in turn, have genetic, social, cultural, familial, and psychological influences (WHO, 2024). Treatment strategies for obesity, such as diet and lifestyle changes, have been shown to be effective in achieving short-term goals; however, most of the weight lost is regained over time (Kheniser et al., 2021). Therefore, it is important to identify effective strategies for weight management that can improve eating behaviors (Warren et al., 2017). Disordered eating is considered a risk factor for the development of obesity or clinical eating disorders, and is related to dysfunctional habits, abnormal beliefs, thoughts, feelings, and behaviors regarding food that may negatively affect physical or emotional health (Alvarenga et al., 2010). In general, the difference between eating disorders and disordered eating behaviors consists of the frequency and severity of the manifestations, as well as a greater impact on weight and health (Tanofsky-Kraff & Yanovski, 2004).

One alternative approach to understanding, modifying, and improving eating behavior is the practice of mindfulness, which has gained significant attention in recent decades for its impact on eating habits and weight management (Jordan et al., 2014; Tapper, 2022).

Mindfulness is defined as a state of awareness in the present moment that allows the mind to focus on the current experience (Bishop et al., 2004; K. Brown et al., 2007). This refers to the ability to pay attention to the body and the mind in the present moment intentionally, without criticism and judgment, which enables an individual to respond in a more adapted way rather than react impulsively (Baer, 2019). According to Bishop et al. (2004), the two-component model of mindfulness includes attentional and attitudinal components. The first, self-regulation of attention, describes an individual’s ability to pay attention to all present-moment experiences (internal or external) such as thoughts, emotions, bodily sensations, and environmental cues. The second refers to a particular orientation towards present-moment experiences and includes attitudes of non-judgment, curiosity, and acceptance.

It has been found that individuals who presented higher levels of mindfulness had a lower likelihood of suffering from obesity, eating disorders, and emotional eating, since the practice of mindfulness refers to a complete awareness of eating and identifying emotional or physiological hunger (Beccia et al., 2018; Demirbas et al., 2021; Godfrey et al., 2015; Mantzios & Wilson, 2015; Özkan & Bilici, 2021; Ruiz-Fuentes & Llorca, 2016). Regarding mindfulness-based interventions, several studies and meta-analyses have concluded that mindful eating may help reduce eating disorder symptoms, binge eating, and emotional eating, although investigations had some limitations such as not including control groups in intervention programs and/or not having rigorous methodology to observe the long-term effects (Godfrey et al., 2015; Lyzwinski et al., 2018; Mason et al., 2016; Mercado et al., 2021; Pepe et al., 2023; Turgon et al., 2019).

Although the benefits of a mindful approach to eating behaviors have been well documented, there is still limited evidence regarding the direct influence of mindful strategies on food intake. Some studies conducted in various countries reported a positive association between mindfulness traits and healthier dietary patterns, including lower total calorie consumption and lower fat and sugar intake in participants (Beshara et al., 2013; Dogan & Tengilimoglu-Metin, 2023; Jordan et al., 2014; Keyte et al., 2020; Mantzios et al., 2018b; Paolassini-Guesnier et al., 2025).

According to previous studies, obesity is highly related to negative emotional states, such as depression, anxiety, stress, anger, or sadness (Aucoin et al., 2021; Hill et al., 2022; Vittengl, 2018). These conditions could contribute to changes in eating habits and lead to an increased (or decreased) appetite with a search for high-calorie comfort food, forming a vicious circle or bidirectional relationship in which an individual is frequently unable to understand the relationship between mood and food consumption (Vittengl, 2018). Additionally, it has been reported that people suffering from obesity have manifested a reduced awareness of eating and low scores of mindful eating (Demirbas et al., 2021; Mantzios et al., 2018a). Regarding the relationship between mindfulness and emotional distress, it has been found that mindful eating was negatively associated with depressive symptoms, anxiety, and uncontrolled or binge eating (Giannopoulou et al., 2020; Metin et al., 2024; Pintado-Cucarella & Rodriguez-Salgado, 2016; Winkens et al., 2018, 2020).

In Mexico, research on mindful eating is scarce, but considering the high prevalence of obesity, this issue is particularly important. Pintado-Cucarella and Rodriguez-Salgado (2016) performed a study on Mexican college students who regularly practiced sports or yoga and who were obese; the Framson et al. (2009) questionnaire was used to assess mindful eating. Results have shown that participants who had less awareness of their eating habits (lower score of mindful eating) were more overweight, more anxious, and had higher levels of negative affect, as well as manifested uncontrolled eating habits and binge eating.

Due to the high prevalence of obesity worldwide and its negative consequences on general health, as well as quality of life, a better understanding of the determining factors of excess weight is required, particularly in young populations. However, there are few studies conducted on this topic among college students (Giannopoulou et al., 2020; Keyte et al., 2020; Mantzios et al., 2018a, 2018b). University life is the transitional stage between adolescence and adulthood, and is of great importance as college students are at risk of making poor dietary choices that can cause significant health problems in the future.

Since cultural identity influences eating behaviors and the meaning of food, the use of specific instruments focused on particular populations is essential. Eating behaviors are influenced by patterns learned within families and society, the unique significance of food in each ethnic group, the evolution of national cuisine over time, and the way each population perceives mindful eating. Olvera-Ruvalcaba et al. (2019) proposed the mindful eating scale for the Mexican population, which contains items directly related to mindful eating and has three questions concerning emotional eating. However, it is relevant to perform studies to identify the associations of this scale with variables related to overweight and obesity, using the complete questionnaire and the questionnaire excluding emotional eating items (as emotional eating may be considered a separate construct).

We hypothesized that individuals who have a lower level of mindful eating, compared with those with a higher level of mindfulness, have higher body fat and unhealthy food consumption. Additionally, the symptoms of emotional distress (depression, anxiety, and stress) are associated with lower levels of mindful eating (Celik-Erden et al., 2023; Dakanalis et al., 2023; Hsu & Forestell, 2021; Konttinen et al., 2019).

The present study aimed to evaluate the association between mindful eating and anthropometric characteristics, body fat, unhealthy food consumption, and emotional distress in college students, using the Mindful Eating Questionnaire including and excluding emotional eating items.

## 2. Materials and Methods

**Figure 1:**
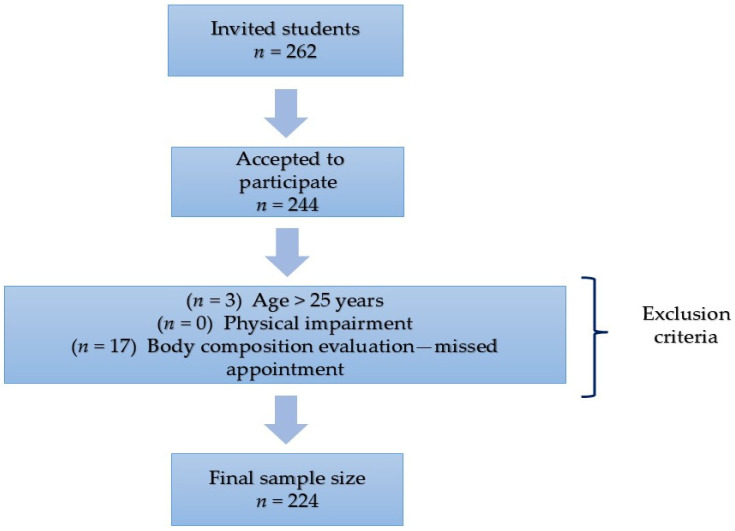
A flow chart of the college student sample selection.

A cross-sectional study was performed at the Metropolitan Autonomous University located in the southeast of Mexico City, Mexico, during the years 2023–2024. The sample size was calculated to detect a difference in mean scores of the Mindful Eating Questionnaire between students with high body mass index (BMI) and those with normal BMI, based on data from a previous Mexican study (Pintado-Cucarella & Rodriguez-Salgado, 2016). A mean difference (delta) of 2.0 was assumed, with a type I error (α) of 0.05 and a power (1−β) of 0.80. The required sample size was estimated to be 192 participants. The sample size calculations were performed using Stata version 15. To ensure sufficient participation, 262 students were invited and 244 accepted. The inclusion criteria were as follows: Regular students of health-related disciplines—Human Nutrition and Medicine. The exclusion criteria were as follows: Students who were older than 25 years old; students with physical disability that did not allow them to participate in anthropometric and body composition measurements; and students who did not complete the questionnaire or did not attend the appointment to perform anthropometric and body composition measurements (20 students). The response rate was 86.6%, considering the initial recruitment and students who did not attend the body composition evaluation appointment. Figure 1 presents the flow chart of the study sample selection. Participants responded to the questionnaire using the Google Drive platform, and it took them approximately 20–30 min. The questionnaire was completed in a university facility.

### 2.1. Anthropometric Measurements

Anthropometric characteristics were assessed in the morning (8 to 9 a.m.). To perform anthropometric evaluation, all participants wore light clothing, were barefoot, and met specific physiological conditions: bowel movement, empty bladder, and a fasting period of at least 8 h. Weight and height were recorded using a Mechanical Physician Scale (capacity 200 kilos, FITH HERGOM Medical, Mexico) with a stadiometer (with an accuracy of 2 mm). Body mass index (BMI weight/height^2^) was categorized according to the World Health Organization criteria (WHO, 2000): underweight <18.5 kg/m^2^, normal weight 18.5 kg/m^2^ to 24.9 kg/m^2^, overweight 25 kg/m^2^ to 29.9 kg/m^2^, and obesity ≥ 30 kg/m^2^. Waist circumference was measured with flexible tape (SECA 201), which was placed around the participants’ waist in the midpoint between the costal edge and iliac crest. The cutoff point was ≥80 cm for females and ≥90 cm for males (Campos-Nonato et al., 2023; Kim et al., 2016).

Body composition assessment was performed using a multifrequency bioelectrical impedance device (In-Body 720 model), which operates with a current between 100 and 500 µA. The device is equipped with eight tactile electrodes—four located on the platform to establish contact with the feet, and four integrated into the hand grips to ensure connection with the fingers, facilitating consistent current flow throughout the body. During the measurement, participants stood barefoot and in an upright (orthostatic) position, as required by standard procedure for bioelectrical impedance analyses. Participants were instructed in advance to wear athletic clothing free of any metallic components. Specifically, female students were advised to wear a tank top instead of a bra with metal clasps to avoid interference with the electrical current. In addition, students were instructed not to wear jewelry or metallic accessories such as earrings, bracelets, or watches on the day of the test. Participants were asked to refrain from engaging in strenuous physical activity or consuming excessive amounts of water during the 24 h prior to the evaluation. On the day of the assessment, they were required to fast for 8 to 12 h, avoid overhydration, and empty their bladder immediately before the procedure. Measurements were conducted in the morning to reduce variability due to circadian changes in body fluid distribution (Kyle et al., 2004). The percentage of body fat was determined using cutoff points of fat percentage for population aged 20–39 years—women 21–32.9% (normal), ≥33% high; men 8–19.9% (normal), ≥20% high—as proposed by Gallagher et al. (2000).

### 2.2. Mindful Eating Instrument

An instrument developed in Mexico with 11 items (Olvera-Ruvalcaba et al., 2019) was applied (Cronbach’s alpha = 0.82), which has two subscales: Mindless Eating (Cronbach’s alpha = 0.84) and Emotional Eating (Cronbach’s alpha = 0.68). The Mindless Eating subscale evaluates the attitude of accepting and non-attaching towards one’s eating behavior with questions such as the following: “I criticize myself when I overeat”; “I feel regret after eating certain foods, for example, junk food, high-fat foods, bread, sweets, among others”; “I think I should eat less”; “When I am eating it is difficult for me to stop worrying about my weight”, among others, as well as eating without distraction: “I think about my plans and projects when I eat”; “I think about my problems when I eat”. The Emotional Eating subscale measures eating behavior related to emotions (“When I feel happy, I eat even if I am not hungry”; “When I feel bored, I eat to distract myself”; “When I feel emotions such as sadness or anger, I eat to feel better”). All questions were in the negative direction (the higher the score, the lower the mindful eating) with a Likert scale from never = 0 to always = 5. In the present study, Cronbach’s alpha of the total scale was 0.86 (Mindless Eating subscale Cronbach’s alpha = 0.86; Emotional Eating subscale = 0.73).

### 2.3. Food Frequency Questionnaire

The assessment of the frequency of food intake was based on the Food Frequency Questionnaire, which consists of 69 items encompassing all groups of foods (Denova-Gutiérrez et al., 2016). The National Survey of Health and Nutrition classified specific foods (the carbohydrate-rich and energy-rich foods) as not recommended for daily consumption for the Mexican population (Gaona-Pineda et al., 2023). Unhealthy foods were categorized into the following groups: sweetened beverages (natural juices, soft drinks, industrially produced flavored drinks or waters with sugar, natural fruit waters with sugar, coffee with sugar, etc.); sweetened dairy drinks (yogurt, Yakult or similar, milk, etc.); fried foods (pork rinds, tortilla chips, potato chips, French fries, fried chicken, etc.); sweets and desserts (candy, pastries, lollipops, cookies, chocolates, biscuits, ice cream, cake or pie, etc.); sweetened cereals (Zucaritas, Corn Flakes, Choco Krispis, etc.); fast food (sandwich, hamburger, pizza, hot dog, etc.); Mexican snacks (sopes, quesadillas, tlacoyos, gorditas, enchiladas, tacos, etc.); processed meats (longaniza or chorizo, sausage or ham high in fat and salt, and/or mortadella, etc.); and alcoholic beverages (beer, a glass of wine, pulque, a shot of tequila, mezcal or another high-alcohol beverage). The frequency of food consumption was assessed using categories of serving size (one time, two times, and three or more times per day), as well as of intake per week (never/almost never, 1 time, 2–3 times, 4 or more times). Intake was defined as not recommended (unhealthy behavior) if participants had consumed ≥1 serving of any food per day ≥1 times per week or for beverages ≥2 times per week.

### 2.4. Depression, Anxiety, and Stress Scale (DASS-21)

To assess depression, anxiety, and stress, the DASS-21 scale was applied, which is the short version of the scale developed by Lovibond and Lovibond (1995) (Antony et al., 1998; T. Brown et al., 1997). The instrument was applied to the Hispanic population and in Mexico (Antúnez & Vinet, 2012; Gurrola et al., 2006). It is a self-reported questionnaire that has three subscales, which allow measuring the presence and intensity of depression symptoms (low positive affectivity: sadness, expectation, personal strength, absence of positivity, enthusiasm, and initiative); anxiety (psychophysiological agitation: breathing, tachycardia, fear, panic, and dry mouth); and stress (negative affectivity: reactivity, susceptibility, nervousness, tiredness, intolerance, and agitation). Cronbach’s alpha of subscales = 0.76, 0.81, and 0.79, respectively; total Cronbach’s alpha = 0.86 (Gurrola et al., 2006). The questionnaire consists of 21 items with 4 possible Likert-type responses: from 0 to 3, the increase in score is related to a greater severity of symptoms.

### 2.5. Statistical Analysis

Categorical variables were described using percentages and frequency distributions to characterize the study group, while means and standard deviations were calculated for continuous variables. Two separate analyses were conducted: the first using the whole Mindful Eating Questionnaire score with 11 questions (ME-11) and the second excluding the emotional eating items (ME-8). Robust regression models were employed to examine the association between mindful eating and anthropometric, and body composition measurements (BMI, waist circumference, and body fat percentage). This statistical technique utilizes estimators less influenced by extreme values, such as the M-estimator or iteratively reweighted least squares, making it well suited for non-normal data. Logistic regression analysis was performed to evaluate the association between the mindful eating score and the frequency of unhealthy food consumption. The results were reported as odds ratios (ORs) with their corresponding 95% confidence intervals (CIs) and *p*-values. To assess the relationship between mindful eating and emotional distress (anxiety, stress, and depression), participants were categorized into tertiles based on their mindful eating scores. The first tertile, representing participants with the lowest scores (indicating a higher level of mindful eating), was used as the reference group in a multinomial logistic regression analysis. The test of hypotheses was set at *p* ≤ 0.05. The statistical package STATA V15 (College Station TX. StataCorp LLC) was used for data analysis.

## 3. Results

A total of 224 college students participated in the study, of whom 173 (77.23%) were women and 51 (22.77%) were men. The age range of the participants was from 19 to 25 years. The mean age of the participants was 21.95 years (*SD* = 2.16). Table 1 summarizes the BMI, waist circumference, body fat percentage, mindful eating, and emotional distress score means. Based on BMI, 86 (38.39%) of the participants were overweight or obese. Additionally, approximately one-third 76 (33.93%) of the students had a high waist circumference, and the number of students with a high percentage of body fat was 116 (51.79%). The mean score for the full version of the Mindful Eating Questionnaire (ME-11) was 26.71 ± 8.62, while the short version excluding emotional eating questions (ME-8) scored 19.85 ± 7.03 (Table 1).

Regarding specific items of the ME questionnaire, more than 60% of participants reported worrying about their weight while eating “sometimes or more often”. Additionally, less than 25% of participants “never” criticized themselves when they overeat. Over two-thirds often regretted consuming junk food, and nearly half reported that they frequently think about their problems while eating. Moreover, about 60% of participants answered that they “sometimes or more often” made plans while eating. Food consumption data are also presented in Table 1. More than 70% of participants reported consuming fast food, fried food, or both two or more times per week.

The results of the logistic regression models examining the association between mindful eating score, anthropometric characteristics, and body composition are presented in Table 2. Higher scores on the ME questionnaire (which indicates a lower level of mindful eating) were associated with higher BMI (*p* < 0.001), waist circumference (*p* < 0.001), and body fat percentage (*p* < 0.001) whether using the full (ME-11) (Table 2a) or short versions (ME-8) (Table 2b) of the questionnaire. The odds ratios (ORs) were comparable between the two scales; for instance, the association with body fat percentage yielded an OR = 1.06 (95% CI: 1.02–1.10) for the full scale (ME-11) and 1.08 (95% CI: 1.03–1.13) for the short version (ME-8). In general, these results suggest that participants with lower levels of mindful eating were more likely to be overweight or obese, and to exhibit higher waist circumference and body fat percentage.

The results of the logistic regression for unhealthy food consumption by mindful eating score are presented in Table 3. Significant associations were identified using the full (ME-11) and short (ME-8) versions. For the ME-11 (Table 3a), lower levels of ME were associated with higher consumption of fried foods (OR = 1.06, 95% CI: 1.02–1.11), sweets and desserts (OR = 1.07, 95% CI: 1.02–1.11), and fast food (OR = 1.05, 95% CI: 1.02–1.09). Similar associations were observed using the ME-8 score (Table 3b). However, no significant associations were found in either version of the ME questionnaire between mindful eating and the consumption of sweetened beverages, sweetened cereals, Mexican snacks, processed meats, or alcohol (*p* > 0.05).

The mental health evaluation included depression, anxiety, and stress symptoms. Table 4 presents the results of the multinomial logistic regression model for ME-11 (Table 4a) and ME-8 (Table 4b) by emotional distress, adjusted for age and sex. Using the ME-11 score and comparing the first with the third tertile, participants with depression symptoms were more likely to be in the third tertile (OR = 1.14, 95% CI (1.05–1.24)). Furthermore, associations were found for anxiety (OR = 1.19, 95% CI (1.09–1.29)) and stress (OR = 1.15, 95% CI (1.05–1.27)). Similar results were found using a multinomial regression model and ME-8 scores (Table 4b). Significant associations were observed for anxiety symptoms in both the second (OR = 1.08, 95% CI (1.01–1.16)) and third tertile (OR = 1.13, 95% CI (1.05–1.23)) using ME-8, as well as in the third tertile of ME-8-based depression symptoms (OR = 1.11, 95% CI (1.02–1.20)) and stress (OR = 1.12, 95% CI (1.02–1.23)). Women were more likely than men to have a low level of mindful eating (Table 4a,b).

## 4. Discussion

This study aimed to identify the associations between mindful eating, overweight/obesity, body fat percentage, unhealthy food consumption, and emotional distress in college students. The findings revealed that lower levels of mindful eating (measured using ME-11 or ME-8) were associated with higher BMI, waist circumference, and body fat percentage, as well as with the consumption of fried foods, sweets, and fast food. Additionally, symptoms of depression, anxiety, and stress were associated with lower levels of mindful eating in both versions of the questionnaire. The results indicated that a considerable number of participants were overweight or obese (38.39%), and a similar percentage had elevated waist circumference. This is consistent with previous studies conducted with Mexican college students (Cruz-Rodríguez et al., 2019; Fernández-Carrasco & López-Ortiz, 2019; Lazarevich et al., 2018; Lorenzini et al., 2015). However, the prevalence was lower than that reported in the National Health and Nutrition Survey for people between 20 and 39 years old (68.0%) (Campos-Nonato et al., 2023; Shamah-Levy et al., 2022). Age and educational level may be contributing factors to this difference.

The consistent results observed between the full version (ME-11) and the short version (ME-8) reinforce the association between mindful eating and anthropometric characteristics, independent of the emotional eating questions. This supports the use of either version of the questionnaire, depending on the research or clinical objectives. Testing the questionnaire against objective measurements provides robust evidence for the link between mindful eating and anthropometric characteristics. Furthermore, incorporating other factors related to obesity, such as the consumption of high-calorie-density foods, and emotional distress (depression, anxiety, and stress symptoms), highlights the multidimensional role of mindfulness in shaping healthier eating behaviors and promoting overall well-being.

In the present study, the participants with higher BMI, waist circumference, and body fat percentage presented a lower level of mindful eating than those with normal parameters. These findings were similar using either version of the questionnaire (ME-11 and ME-8), suggesting that mindful eating independently influences anthropometric characteristics regardless of emotional eating items. The results coincide with previous investigations; in Mexico, Pintado-Cucarella and Rodriguez-Salgado (2016) reported that college students who had less awareness of their eating habits were more frequently overweight than participants who practice yoga or sport at a professional or recreational level.

In the USA, in college students, a relationship between mindful eating score and BMI was observed (Moor et al., 2013). In line with these findings, studies performed on adults from the UK and Turkey have identified an association between mindful eating and overweight/obesity (Demirbas et al., 2021; Hinton et al., 2024).

Another finding of the present study was that participants with more frequent consumption of fried foods, sweets and desserts, and fast food had lower levels of mindful eating, using both versions of the questionnaire (ME-11 and ME-8). This association highlights how limited awareness and attention while eating can contribute to the selection of less nutritious food options. However, no significant association was observed between mindful eating and the consumption of sweetened beverages, processed meats, or alcohol, suggesting that other factors may influence these behaviors in college students (Hilger-Kolb et al., 2017; Muñoz-Rodríguez et al., 2021).

Although there are few reports about mindful eating and specific food consumption in college students (Mantzios et al., 2018a, 2018b) and in other population groups (Beshara et al., 2013; Dogan & Tengilimoglu-Metin, 2023; Paolassini-Guesnier et al., 2025), a positive association was found between higher mindfulness in eating and lower total calorie intake, fat and sugar intake, serving size of energy-dense foods, motives to eat palatable food, and ultra-processed food consumption.

Previous studies conducted in different countries have documented that college students have poor eating habits that can lead to weight gain, such as consumption of pastry snacks, cold meats, fried foods, fast food, carbonated soft sugary drinks daily or almost daily, as well as skipping breakfast and high consumption of alcohol (Durán et al., 2017; Gradidge & Cohen, 2018; Lazarevich et al., 2018; López et al., 2019; Olatona et al., 2018; Omage & Omuemu, 2018). Additionally, most undergraduate students eat at college facilities where healthy food options are limited and typically more expensive than other types of food. Many of them live away from their families and adapt to new social norms by imitating the habits of their peers. They also learn to manage their time, cope with high stress levels, take on new financial responsibilities, and develop independence and decision-making skills (Hilger-Kolb et al., 2017).

Regarding emotional distress, in the present study, participants with higher depression, anxiety, and stress scores have shown lower levels of mindful eating, which is consistent with previous studies performed in different countries among college students (Giannopoulou et al., 2020; Metin et al., 2024; Pintado-Cucarella & Rodriguez-Salgado, 2016). In these situations, negative emotions can reduce awareness of eating and affect the capacity to observe the necessities of their own body, as well as lead to increased appetite and unhealthy eating habits that can be a compensatory mechanism to improve mood.

The bidirectional relationship between obesity and depression has been reported previously (Fulton et al., 2021; Vittengl, 2018), and anxiety is considered the most frequent condition related to unhealthy dietary patterns. An analysis of 1541 articles performed by Aucoin et al. (2021) revealed an association between higher levels of anxiety and a high-fat diet, high intake of sugar and refined carbohydrates, as well as “unhealthy” dietary patterns. Stress can also lead to the disruption of eating behaviors. In a systematic review and meta-analysis, stress was associated with an increased consumption of unhealthy foods (Hill et al., 2022).

During university life, students may change their dietary habits and eating behaviors, which can be healthy or unhealthy. It is assumed that mindfulness assessment and training can enable individuals to increase the ability to pay attention in a specific moment and to relate to this moment in a non-evaluative and accepting manner, which allows them to respond in a more adapted way rather than react in an impulsive manner (Baer, 2019). Therefore, mindfulness training could complement traditional educational programs and help students better understand how mindful eating influences eating behavior, among other factors. Additionally, educational programs aimed at reducing stress and negative effects in the promotion of healthy eating could be the best goal for individuals who are at risk of overeating in response to emotions. Furthermore, the topic of mindful eating should be included in the curriculum of health-related disciplines.

One of the limitations of this study is that the sample consisted of health science students, a specific population, which means the results may not be directly generalized to students of other disciplines. Despite this, they represent an important group given their future as health educators and role models. On the other hand, the availability and overconsumption of ultra-processed foods, mindless eating, and emotional distress could be present in other young adults, making our findings relevant for different population groups. Moreover, the cross-sectional design of the study restricts the ability to determine causal relationships. Additionally, response bias may have occurred due to the use of self-reported questionnaires, as individuals may interpret the questions differently. Also, biases of social desirability may arise when respondents provide answers that they believe are more socially acceptable or healthier rather than accurately reporting their actual eating habits. However, to mitigate these biases, the participants were assured of data confidentiality in the informed consent, which was obtained from all students involved in the study.

This study examined the relationship between mindful eating and several factors, including BMI, body fat percentage, unhealthy eating habits, and emotional problems. The fact that mindful eating scores were significantly associated with these characteristics supports the validity of the questionnaire. Additionally, these results align with findings from previous research. Further studies, particularly longitudinal research, are needed to better understand the relationships between mindful eating, dietary intake, and emotional distress. Additionally, it is essential to identify mediating mechanisms associated with the relationship between mindful eating and obesity.

## 5. Conclusions

In the present study, mindful eating was associated with body mass index, eating habits, and emotional distress, which suggests that mindfulness could be used in multidisciplinary educational and intervention programs to promote healthy eating.

The consistent results observed between the full version (ME-11) and the short version (ME-8) reinforce the association of mindful eating with the study variables, independent of emotional eating, which allows the use of the questionnaire for different types of research or intervention programs.

Mindful eating is important to consider in weight management to increase awareness of eating and reduce the impact of negative emotions on eating behavior, offering a dual benefit for both psychological well-being and dietary patterns.

The findings of the present study underscore the importance of promoting mindful eating practices as a potential strategy to address multiple health concerns in Mexican college students such as improving body composition, reducing the consumption of unhealthy food, and mitigating symptoms of depression, anxiety, and stress. Given the high prevalence of overweight, obesity, unhealthy eating behaviors, and emotional distress challenges in this population, integrating mindful eating programs into student health initiatives could have substantial public health benefits.

## Figures and Tables

**Table 1:** Descriptive characteristics of participants (*n* = 224).

Variable	Mean (± SD) or *n* (%)
Age (years)	21.95 ± 2.16
Sex	
Female	173 (77.23)
Male	51 (22.77)
Body Mass Index (kg/m^2^)	
(mean ± SD)	24.21 ± 3.88
Normal (≥18.5 and <25)	138 (61.61)
Overweight/obesity (≥25)	86 (38.39)
Waist Circumference (cm) (cutoff values females ≥80, males ≥90)	
Normal	148 (66.07)
High	76 (33.93)
Body Fat (%) (cutoff values females ≥ 33, males ≥ 20)	
Normal	108 (48.21)
High	116 (51.79)
Mindful Eating Score (ME-11) (mean ± SD)	26.71 ± 8.62
Mindful Eating Score (excluding emotional eating items, ME-8) (mean ± SD)	19.85 ± 7.03
Emotional Distress (DASS-21) ^1^ (mean ± SD)	
Depression score	16.27 ± 4.59
Anxiety score	17.65 ± 4.66
Stress score	14.21 ± 3.82
Food Consumption	
Sweetened Beverages	
≥2 times per week	98 (43.75)
<2 times per week	126 (56.25)
Sweetened Dairy Drinks	
≥2 times per week	61 (27.23)
<2 times per week	163 (72.77)
Fried Foods	
≥1 time per week	173 (77.23)
<1 time per week	51 (22.77)
Sweets and Desserts	
≥1 time per week	168 (75.00)
<1 time per week	56 (25.00)
Sweetened Cereals	
≥1 time per week	60 (26.79)
<1 time per week	164 (73.21)
Fast Food	
≥1 time per week	138 (61.61)
<1 time per week	86 (38.39)
Mexican Snacks	
≥1 time per week	149 (66.52)
<1 time per week	75 (33.48)
Processed Meat	
≥1 time per week	148 (66.07)
<1 time per week	76 (33.93)
Alcohol	
≥2 times per week	80 (35.71)
<2 times per week	144 (64.29)

^1^ DASS-21: Depression, Anxiety, and Stress Scale.

**Table 2:** (**a**). Results of logistic regression for anthropometric characteristics and body fat percentage by mindful eating scores, including all items of questionnaire (ME-11). (**b**). Results of logistic regression models for anthropometric characteristics and body fat percentage by mindful eating score, excluding emotional eating items (ME-8).

**(a) ME-11**			
	**Outcome Variable**	**OR (95% CI) ^1^**	***p***
	Body Mass Index ^3^		
Mindful eating score (ME-11) ^2^		1.08 (1.04–1.12)	<0.001
	Waist Circunference ^4^		
Mindful eating score (ME-11) ^2^		1.09 (1.04–1.13)	<0.001
	Body Fat (%) ^5^		
Mindful eating score (ME-11) ^2^		1.06 (1.02–1.10)	<0.001
**(b) ME-8**			
	**Outcome Variable**	**OR (95% CI) ^1^**	***p***
	Body Mass Index ^3^		
Mindful eating score (ME-8) ^2^		1.10 (1.05–1.15)	<0.001
	Waist Circunference ^4^		
Mindful eating score (ME-8) ^2^		1.11 (1.06–1.16)	<0.001
	Body Fat (%) ^5^		
Mindful eating score (ME-8) ^2^		1.98 (1.03–1.13)	<0.001

^1^ OR of the logistic regression model adjusted by sex and age. ^2^ All questions are in the negative direction (the higher the score, the lower the mindful eating level). ^3^ Cutoff point for overweight/obesity: BMI ≥ 25. ^4^ Cutoff point for waist circumference: ≥80 cm for females and ≥90 cm for males ^5^ Cutoff points for body fat percentage: ≥33% for females and ≥20% for males.

**Table 3:** (**a**). The results of the logistic regression models for the frequency of unhealthy food consumption by mindful eating score—whole version (ME-11). (**b**). The results of the logistic regression models for the frequency of unhealthy food consumption by mindful eating score, excluding emotional eating items (ME-8).

**(a) ME-11**			
	**Outcome Variable**	**OR (95% CI) ^1^**	***p***
	Sweetened Beverages ^3^		
Mindful eating score (ME-11) ^2^		1.01 (0.98–1.05)	0.468
	Sweetened Dary Drinks ^3^		
Mindful eating score (ME-11) ^2^		1.02 (0.99–1.06)	0.224
	Fried Food ^4^		
Mindful eating score (ME-11) ^2^		1.06 (1.02–1.11)	0.005
	Sweets and Desserts ^4^		
Mindful eating score (ME-11) ^2^		1.07 (1.02–1.11)	0.003
	Sweetened Cereals ^4^		
Mindful eating score (ME-11) ^2^		1.01 (0.97–1.05)	0.656
	Fast Food ^4^		
Mindful eating score (ME-11) ^2^		1.05 (1.02–1.09)	0.003
	Mexican Snacks ^4^		
Mindful eating score (ME-11) ^2^		1.00 (0.97–1.04)	0.867
	Procesesed Meat ^4^		
Mindful eating score (ME-11) ^2^		1.00 (0.98–1.04)	0.610
	Alcohol ^3^		
Mindful eating score (ME-11) ^2^		1.03 (0.99–1.06)	0.126
**(b) ME-8**			
	**Outcome Variable**	**OR (95% CI) ^1^**	***p***
	Sweetened Beverages ^3^		
Mindful eating score (ME-8) ^2^		1.01 (0.97–1.05)	0.621
	Sweetened Dary Drinks ^3^		
Mindful eating score (ME-8) ^2^		1.02 (0.97–1.06)	0.457
	Fried Food ^4^		
Mindful eating score (ME-8) ^2^		1.06 (1.01–1.11)	0.024
	Sweets and Desserts ^4^		
Mindful eating score (ME-8) ^2^		1.06 (1.01–1.12)	0.026
	Sweetened Cereals ^4^		
Mindful eating score (ME-8) ^2^		1.00 (0.96–1.05)	0.974
	Fast Food ^4^		
Mindful eating score (ME-8) ^2^		1.05 (1.01–1.10)	0.020
	Mexican Snacks ^4^		
Mindful eating score (ME-8) ^2^		1.00 (0.96–1.04)	0.897
	Procesesed Meat ^4^		
Mindful eating score (ME-8) ^2^		1.00 (0.97–1.04)	0.938
	Alcohol ^3^		
Mindful eating score (ME-11) ^2^		1.02 (0.98–1.06)	0.429

^1^ OR of the logistic regression model adjusted by sex and age. ^2^ All questions are in the negative direction (the higher the score, the lower the mindful eating level). ^3^ Consumption frequency ≥ 2 times per week; ^4^ consumption frequency ≥ 1 times per week.

**Table 4:** (**a**). Results of logistic regression for mindful eating scores (tertiles)—full version—by depression, anxiety, and stress scores (DASS-21) ^1^ (**b**). Results of logistic regression for mindful eating scores (tertiles)—excluding emotional eating items—by depression, anxiety, and stress scores (DASS-21) ^1^.

**(a) ME-11**			
**Mindful Eating, Including All Items of the Questionnaire (ME-11) ^2^** **Outcome Variable**	**Predictor** **Variables**	**OR (95% CI)**	***p***
Low Score (Tertile 1, reference)			
Medium Score (Tertile 2)	Depression Score	1.04 (0.97–1.12)	0.253
	Age	1.04 (0.94–1.15)	0.466
	Sex (Female = 1)	1.51 (0.74–3.09)	0.253
High Score (Tertile 3)	Depression Score	1.14 (1.05–1.24)	<0.001
	Age	1.03 (0.92–1.14)	0.642
	Sex (Female = 1)	5.91 (2.22–15.73)	<0.001
Medium Score (Tertile 2)	Anxiety Score	1.06 (0.99–1.14)	0.112
	Age	1.04 (0.94–1.16)	0.425
	Sex (Female = 1)	1.49 (0.73–3.05)	0.273
High Score (Tertile 3)	Anxiety Score	1.19 (1.09–1.29)	<0.001
	Age	1.04 (0.93–1.16)	0.488
	Sex (Female = 1)	5.89 (2.17–15.97)	<0.001
Medium Score (Tertile 2)	Stress Score	1.06 (0.98–1.16)	0.150
	Age	1.04 (0.94–1.15)	0.473
	Sex (Female = 1)	1.44 (0.71–2.95)	0.315
High Score (Tertile 3)	Stress Score	1.15 (1.05–1.27)	0.003
	Age	1.03 (0.92–1.14)	0.631
	Sex (Female = 1)	5.24 (1.98–13.86)	<0.001
**(b) ME-8**			
**Mindful Eating Score, Excluding Emotional Eating Items of the Questionnaire (ME-8) ^2^** **Outcome Variable**	**Predictor** **Variable**	**OR (95% CI)**	***p***
Low Score (Tertile 1, reference)			
Medium Score (Tertile 2)	Depression Score	1.06 (0.99–1.14)	0.078
	Age	1.07 (0.96–1.19)	0.241
	Sex (Female = 1)	2.51 (1.21–5.22)	0.014
High Score (Tertile 3)	Depression Score	1.11 (1.02–1.20)	0.011
	Age	1.04 (0.93–1.17)	0.490
	Sex (Female = 1)	5.90 (2.24–15.54)	<0.001
Medium Score (Tertile 2)	Anxiety Score	1.08 (1.01–1.16)	0.026
	Age	1.07 (0.96–1.20)	0.207
	Sex (Female = 1)	2.45 (1.18–5.12)	0.017
High Score (Tertile 3)	Anxiety Score	1.13 (1.05–1.23)	0.002
	Age	1.05 (0.93–1.18)	0.404
	Sex (Female = 1)	5.74 (2.17–15.22)	<0.001
Medium Score (Tertile 2)	Stress Score	1.10 (1.01–1.20)	0.031
	Age	1.07 (0.96–1.19)	0.243
	Sex (Female = 1)	2.35 (1.13–4.90)	0.023
High Score (Tertile 3)	Stress Score	1.12 (1.02–1.23)	0.017
	Age	1.04 (0.93–1.17)	0.494
	Sex (Female = 1)	5.40 (2.24–15.54)	<0.001

^1^ DASS-21: Depression, Anxiety, and Stress Scale; ^2^ high scores indicate less mindful eating.

## Data Availability

The data are available from the authors upon reasonable request.

## Abbreviations

The following abbreviations are used in this manuscript:

ME	Mindful Eating
BMI	Body Mass Index
ME-11	Mindful Eating Questionnaire, 11 items
ME-8	Mindful Eating Questionnaire, 8 items
EE	Emotional Eating
